# Area-Efficient Mapping of Convolutional Neural Networks to Memristor Crossbars Using Sub-Image Partitioning

**DOI:** 10.3390/mi14020309

**Published:** 2023-01-25

**Authors:** Seokjin Oh, Jiyong An, Kyeong-Sik Min

**Affiliations:** School of Electrical Engineering, Kookmin University, Seoul 02707, Republic of Korea

**Keywords:** area-efficient mapping, convolutional neural networks, memristor crossbars, sub-image partitioning

## Abstract

Memristor crossbars can be very useful for realizing edge-intelligence hardware, because the neural networks implemented by memristor crossbars can save significantly more computing energy and layout area than the conventional CMOS (complementary metal–oxide–semiconductor) digital circuits. One of the important operations used in neural networks is convolution. For performing the convolution by memristor crossbars, the full image should be partitioned into several sub-images. By doing so, each sub-image convolution can be mapped to small-size unit crossbars, of which the size should be defined as 128 × 128 or 256 × 256 to avoid the line resistance problem caused from large-size crossbars. In this paper, various convolution schemes with 3D, 2D, and 1D kernels are analyzed and compared in terms of neural network’s performance and overlapping overhead. The neural network’s simulation indicates that the 2D + 1D kernels can perform the sub-image convolution using a much smaller number of unit crossbars with less rate loss than the 3D kernels. When the CIFAR-10 dataset is tested, the mapping of sub-image convolution of 2D + 1D kernels to crossbars shows that the number of unit crossbars can be reduced almost by 90% and 95%, respectively, for 128 × 128 and 256 × 256 crossbars, compared with the 3D kernels. On the contrary, the rate loss of 2D + 1D kernels can be less than 2%. To improve the neural network’s performance more, the 2D + 1D kernels can be combined with 3D kernels in one neural network. When the normalized ratio of 2D + 1D layers is around 0.5, the neural network’s performance indicates very little rate loss compared to when the normalized ratio of 2D + 1D layers is zero. However, the number of unit crossbars for the normalized ratio = 0.5 can be reduced by half compared with that for the normalized ratio = 0.

## 1. Introduction

Memristor crossbars can be used for computing MAC (Multiplication and Accumulation) operation in their memory array, because the memristor’s current can be calculated with Ohm’s law of ‘i = G × v’ [1,2,3,4]. Here, ‘G’ is the memristor’s conductance in the crossbar, which can be programmed by applying voltage or current pulse [5,6,7,8]. In Ohm’s law, ‘i’ and ‘v’ are the memristor’s current and voltage, respectively. If ‘v’ is applied as an input voltage to the memristor, the memristor’s current, ‘i’, can be thought of as the multiplication result of memristor’s conductance ‘G’ and input voltage ‘v’. If many input voltages are applied to the crossbar’s rows simultaneously, the crossbar’s column current can be thought as the summation of multiplications, which can be calculated with $i_{col,j}=\underset{m}{\sum }G_{i}v_{row,i}$. In this equation, ‘i_col,j_‘ is column(j)’s current. ‘m’ is the number of rows in the crossbar. ‘G_i_’ and ‘v_row,i_’ are memristor(i)’s conductance and input voltage(i), respectively. ‘j’ and ‘i’ are the indices of crossbar’s column and row, respectively.

By doing so, the vector matrix multiplication can be calculated physically using the memristor crossbar’s current–voltage relationship, where each column current is the MAC operation result of the corresponding column. The physical MAC calculation by memristor crossbars can consume smaller energy than the conventional computing using CMOS digital circuits [9,10,11]. Moreover, if memristors can store multi-values such as 7-8 bits, the layout area for the physical computing by memristors can be much smaller than the digital CMOS circuits for performing the MAC operation. These advantages of low energy and small layout area of computing are very beneficial for implementing neural network hardware especially at edge devices such as Internet of Things (IoT) devices [12,13].

**Figure 1:**
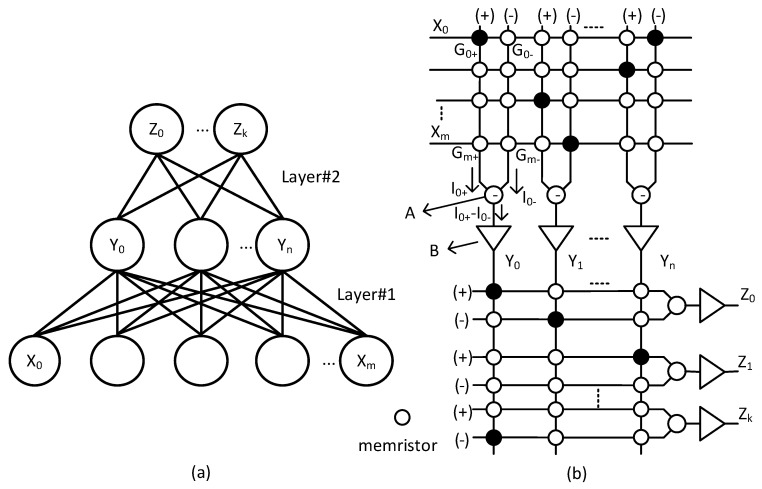
(**a**) The block diagram of artificial neural networks with input, hidden, and output neurons (**b**) The memristor crossbars for implementing the neural networks.

Figure 1a shows a conceptual block diagram of artificial neural networks. In Figure 1a, the input neurons are connected to the hidden ones through synapses. Each synaptic connection has its weight. Similarly, the hidden neurons are connected to the output ones though synapses, too. Here, the input, hidden, and output neurons are represented with X, Y, and Z, respectively. ‘m’, ‘n’, and ‘k’ are the numbers of input, hidden, and output neurons, respectively. The neural networks in Figure 1a can be realized by memristor crossbars, as indicated in Figure 1b. More specifically, layer #1 between the input and hidden neurons in Figure 1a is implemented by the upper crossbar in Figure 1b. Here, the input and hidden neurons can be designed by CMOS analog circuits. The lower crossbar in Figure 1b is for layer #2 between the hidden and output neurons in Figure 1a. The two columns represented with (+) and (-) in Figure 1b can calculate both positive and negative synaptic weights in neural networks. The two columns in memristor crossbars are needed because the synaptic weights can be both positive and negative. Each synaptic weight in the neural networks in Figure 1a can be realized by each memristor’s conductance in the crossbars in Figure 1b.

Explaining the MAC calculation by memristor crossbars more in detail, in Figure 1b, G_0+_ and G_0-_ represent the memristor’s conductance on plus and minus columns, respectively, for ‘row #0′. X_0_ is input voltage applied to ‘row #0′. Similarly, G_m+_ and G_m-_ are the memristor’s conductance for ‘row #m’. X_m_ is the input voltage to ‘row #m’ in Figure 1b. Here, I_0+_ can be calculated with $G_{0+}X_{0}+\|+G_{m+}X_{m}$. I_0-_ is $G_{0-}X_{0}+\|+G_{m-}X_{m}$. The difference of I_0+_ and I_0-_ is calculated with $I_{0+}-I_{o-}$ by circuit (A). The calculated $I_{0+}-I_{o-}$ enters the voltage amplifier (B), where Y_0_ is obtained and delivered to the next crossbar. Here, $G_{0+}-G_{o-}$ can be regarded as a synaptic weight. If G_0+_ is larger than G_0-_, the weight is positive. If G_0+_ is smaller than G_0-_, the weight is negative. Similarly, $G_{m+}-G_{m-}$ can be regarded as the other synaptic weight. By doing so, both positive and negative weights can be represented using the (+) and (-) columns as shown in Figure 1b [14].

One thing to consider in implementing the neural networks in Figure 1a by the crossbars in Figure 1b is that the crossbar’s size should be limited due to the line resistance problem [15,16,17,18,19]. If the crossbar’s size is too large and the line resistance becomes comparable to the LRS (Low Resistance State) value, the MAC calculation accuracy can be degraded significantly [15,19,20]. For example, the recently fabricated 40 nm RRAM array has 128 × 128 cells, and the line resistance per cell was measured ~1.1Ω. Thus, the total line resistance can be as large as 141Ω. In this case, the voltage drop on line resistance can be negligible if the memristor’s LRS (Low Resistance State) is as large as 10KΩ [19]. If the crossbar’s size becomes as large as, for example, 1024 × 1024, the line resistance becomes ~1.1KΩ. This large line resistance can degrade the MAC calculation accuracy severely if the LRS is as small as a few kilo ohms.

From the previous publications about the experimental line resistance measured from academia and industries, the crossbar’s size can be 128 × 128 or 256 × 256 [19,21]. Unfortunately, however, these crossbar’s sizes are too small to process most of the deep-learning image datasets such as CIFAR-10 (32 × 32 × 3), IMAGENET (224 × 224 × 3), etc. [22,23]. Thus, a full image with a very large number of pixels should be divided into small sub-images for processing them in unit crossbars, where the crossbar’s size can be defined as 128 × 128 or 256 × 256. By doing so, the line resistance problem can be avoided in the sub-image partitioning instead of using the full image.

One of the important operations used in neural networks is convolution operation. Similarly with the previous discussion, for performing the convolution by memristor crossbars, the full-image convolution should be partitioned into several sub-image convolution blocks. By doing so, each sub-image convolution can be mapped to the unit crossbar instead of the large-size crossbar [24]. In this paper, the crossbar’s size is assumed as 128 × 128 and 256 × 256 to avoid the line resistance problem caused from large-size crossbars [19,21]. When the sub-image convolution is mapped to unit crossbars, the overlapping overhead can be caused from the borderline pixels between two neighboring sub-images. This is because the borderline pixels should be involved in both the convolution calculations of two neighboring sub-images to avoid the edge effect. The overlapping between two neighboring sub-images due to the borderline pixels can increase the number of crossbar’s rows needed in the sub-image convolution.

In this paper, to mitigate the overlapping overhead caused from the mapping of sub-image convolution to unit crossbars, various convolution schemes using 3D, 2D, and 1D kernels are investigated and compared, because the overlapping overhead can be different for the different convolutions with 3D, 2D, and 1D kernels. The 3D kernel is composed of width, length, and height. In the sub-image convolution with 3D kernels, the overlapping overhead becomes significantly large, because two neighboring sub-images can share many borderline pixels in the both lateral and vertical dimensions, as will be explained in the next section. The 2D kernel composed of only width and length is used in depthwise convolution, where the overlapping can be found only in the lateral direction, not in the vertical direction. By doing so, the overlapping overhead of 2D convolution can be smaller than the convolution with 3D kernel. The 1D kernel can perform pointwise convolution. In this case, no overlapping overhead can occur in the both lateral and vertical directions. Of course, the overlapping overhead due to the 1D kernel is the smallest among the three kernels. Based on the comparative study on various convolution schemes with the 3D, 2D, and 1D kernels, an area-efficient mapping method of sub-image convolution to unit crossbars is proposed to minimize the overlapping overhead due to the borderline pixels shared between two neighboring sub-images in the following section. In Section 3, the simulation results are shown and discussed to verify that the proposed mapping method of sub-image convolution can succeed in improving the overlapping overhead due to borderline pixels. Finally, we summarize this paper in Section 4.

## 2. Method

**Figure 2:**
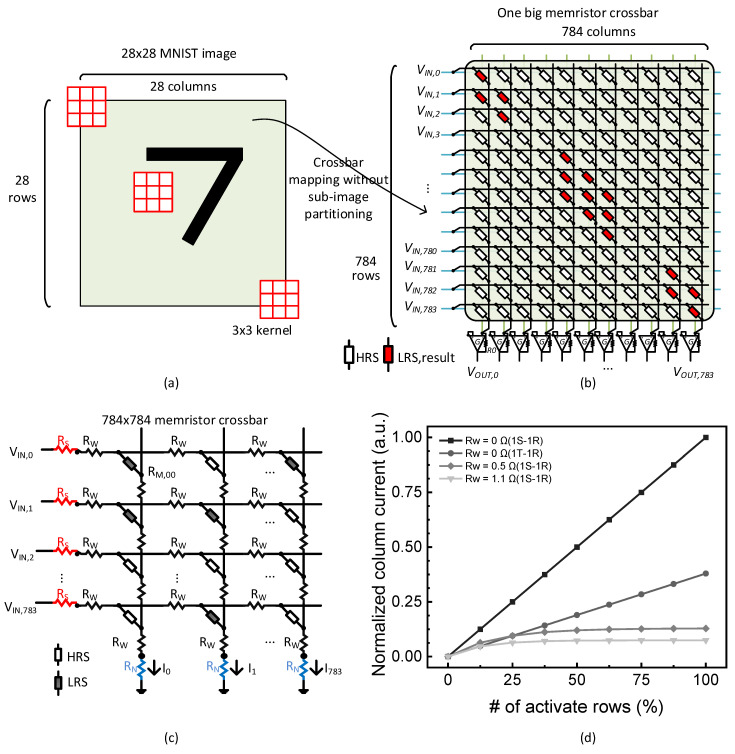
(**a**) The convolution of 28 × 28 MNIST image with 3 × 3 kernel without using the sub-image partitioning. (**b**) The memristor crossbar for the full-image convolution without using the sub-image partitioning. (**c**) The crossbar circuit with parasitic resistance such as source, line, and neuron resistance. (**d**) The normalized column current with increasing the number of active rows (%), for 1S-1R and 1T-1R cells. Here, R_W_ means line resistance per cell and the crossbar’s column has 784 cells per column. When R_W_ = 0.5 Ω and R_W_ = 1.1 Ω, the normalized column currents seem to saturate rapidly with increasing the percentage active rows over 25%. It means the MAC calculation accuracy is degraded very much when R_W_ is not zero.

As mentioned in the previous section, in this paper, we try to propose an area-efficient mapping method of neural network’s convolution to crossbars. To do so, first, we consider that the convolution is mapped to one big crossbar without sub-image partitioning. Figure 2a shows the convolution of a 28 × 28 MNIST image with a 3 × 3 kernel without using the sub-image partitioning. In this figure, the 3 × 3 kernel is represented in red. For performing the convolution, the 3 × 3 kernel is moved from the top-left to the bottom-right in the 28 × 28 input image to calculate features from the input image. Figure 2b shows a large-size memristor crossbar for performing the full-image convolution without the use of sub-image partitioning. Here, the number of crossbar’s rows is the same with the number of input pixels involved in the convolution. Similarly, the number of crossbar’s columns is equal to the number of output pixels calculated from the convolution. Thus, for performing the convolution of 28 ×28 MNIST image with a 3 × 3 kernel, the crossbar’s row and column numbers should be 784 and 784, respectively, as indicated in Figure 2b.

One problem of the memristor crossbar in Figure 2b is that the crossbar’s line resistance can be very large because the crossbar’s size is large. As mentioned earlier, the line resistance is increased more as the crossbar’s size becomes larger. The large line resistance can degrade the crossbar’s MAC calculation accuracy significantly. If so, the MAC calculation result from the memristor crossbar can be different from the ideal MAC calculation. Figure 2c shows a memristor crossbar circuit with parasitic resistance. Here, R_S_, R_W_, and R_N_ are the parasitic source, line, and neuron resistance, respectively [15]. V_IN,0_ is input voltage applied to ‘row #0′. I_0_ is the column current from ‘column #0′. In Figure 2c, the input voltages such as V_IN,0_ are applied to the crossbar’s rows. The currents generated by the crossbar’s columns can be thought of as the MAC results calculated physically from the memristor crossbar.

Figure 2d indicates that the MAC calculation accuracy is affected significantly due to the parasitic resistance such as R_W_. Here, the crossbar is assumed to have 784 cells per column, as shown in Figure 2c. The R_W_ means line resistance per cell. If the column has 784 cells and R_W_ = 1.1 Ω, the total line resistance becomes as large as 862 Ω. In this figure, the normalized column current means the MAC calculation result is plotted with increasing the percentage number of active rows among 784 rows. The ‘active rows’ means the row’s input voltage is high. If the percentage number of active rows is 50%, 392 rows are applied by high voltage and the other 392 are driven by 0V, among the total 784 inputs. Here, 1T-1R means the crossbar composed of 1 transistor and 1 memristor. 1S-1R is the array made of a self-rectifying memristor. For 1T-1R, the effective LRS resistance considering both LRS and transistor’s ON resistance is assumed to be 26.3 KΩ in the circuit simulation of Figure 2d. The effective HRS resistance considering both HRS and the transistor’s ON resistance can be the same with HRS = 1 MΩ, because the HRS is much larger than the transistor’s ON resistance, as explained later in Section 3. In 1S-1R, the selector may be united with the memristor not using an external transistor as the selector.

When R_W_ = 0 Ω, the normalized column current seems very linear upon increasing the percentage of active rows among 784 rows for both 1S-1R and 1T-1R cells. It indicates clearly that the MAC calculation accuracy is not degraded regardless of 1S-1R and 1T-1R cells. However, when R_W_ = 0.5 Ω and R_W_ = 1.1 Ω, the normalized column currents seem to saturate rapidly with increasing the percentage active rows over 25%. It means the MAC calculation accuracy is degraded very much when R_W_ is not zero. If R_W_ becomes larger, the MAC calculation accuracy becomes degraded more, as shown in Figure 2d. From the circuit simulation of MAC calculation by the crossbar’s column current, the line resistance shows that it can degrade MAC calculation accuracy significantly. Based on the analysis of Figure 2d, we discuss how to mitigate the line resistance problem in memristor crossbars in the following paragraphs.

**Figure 3:**
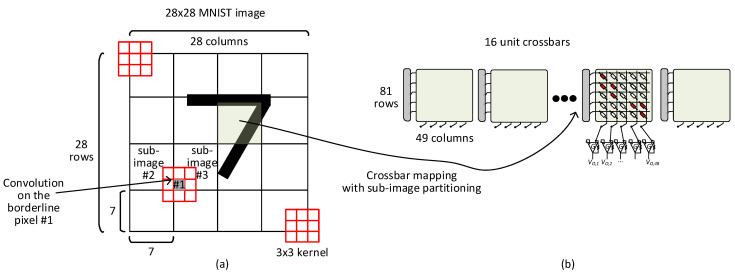
(**a**) The convolution of 28 × 28 MNIST image with 3 × 3 kernel using the sub-image partitioning. (**b**) The memristor crossbar for the sub-image convolution using the sub-image partitioning. Here, the borderline rows and columns between two neighboring sub-images are included in the crossbar’s row number of 81(=9 × 9). When the crossbar’s column number is calculated, only the number of output pixels of the convolution should be considered. By doing so, the crossbar’s column number is equal to the sub-image size of 49(=7 × 7).

For overcoming the large line resistance problem due to large-size crossbars, the MNIST image should be partitioned into several sub-images. For example, the 28 × 28 MNIST image can be divided into sixteen 7 × 7 sub-images, as indicated in Figure 3a. Here, each 7 × 7 sub-image convolution can be mapped to memristor crossbars with much smaller size than 784 × 784, as shown in Figure 3b. As explained earlier, the number of crossbar’s rows is the same with the number of input pixels involved in the convolution. Similarly, the number of crossbar’s columns is equal to the number of output pixels calculated from the convolution. By doing so, the crossbar’s row and column numbers for 7 × 7 sub-image convolution can be calculated with 81(=9 × 9) and 49(=7 × 7), respectively, as shown in Figure 3b.

Here, it should be noted that the borderline pixels overlapping between two neighboring sub-images should be considered in counting the crossbar’s row number as many as 81(=9 × 9). In Figure 3a, if we look at the borderline pixel ‘#1′ between two neighboring sub-images of ‘#2′ and ‘#3′, the borderline pixel ‘#1′ is involved in the convolution for both the sub-images of ‘#2′ and ‘#3′. This overlapping overhead due to the borderline pixels can increase the crossbar’s row number. On the contrary, when the crossbar’s column number is considered, only the number of output pixels calculated from the convolution should be counted. By doing so, the crossbar’s column number is equal to the sub-image size of 49(=7 × 7).

Comparing the crossbar size for between the full-image convolution (784 × 784) in Figure 2a and sub-image convolution (81 × 49) in Figure 3a indicates clearly that the crossbar’s size of sub-image convolution can be ~10x smaller than that of full-image convolution. This crossbar’s size reduction can decrease the line resistance by 90%, resulting in improving the MAC calculation accuracy significantly.

As explained earlier, the memristor’s size should be very large for performing the full-image convolution. If the memristor’s size is very large, the line resistance can be very large, too. If so, the MAC calculation accuracy can be degraded significantly. To avoid the line resistance problem, the full image should be partitioned into smaller sub-images. Each sub-image convolution can be performed by each unit crossbar, where the unit crossbar’s line resistance can be much smaller than the crossbar of full-image convolution. However, when the sub-image convolution is mapped to small-size unit crossbars, the borderline pixels overlapping between two neighboring sub-images can cause the overlapping overhead, because the crossbar’s row number is increased.

**Figure 4:**
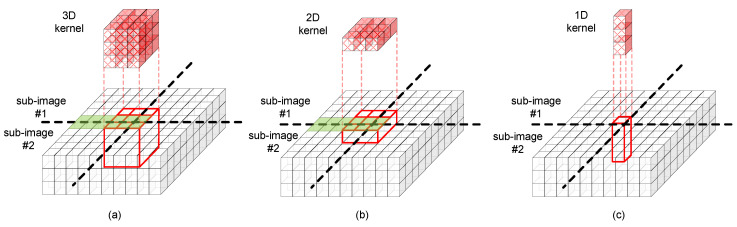
(**a**) The sub-image convolution with 3D kernels. (**b**) The sub-image convolution with 2D kernels. (**c**) The sub-image convolution with 1D kernels.

The overlapping overhead mentioned just earlier can be different for various convolution schemes with different kernels. Figure 4a–c show the sub-image convolution with 3D, 2D, and 1D kernels, respectively. Here, the borderline pixels overlapping between the two neighboring sub-images of ‘#1′ and ‘#2′ are shown in green. As shown in Figure 4a, the sub-image convolution with 3D kernels can increase the overlapping overhead very severely, because the overlapping due to 3D kernels can occur in both the lateral and vertical directions. Figure 4b indicates the overlapping overhead due to 2D kernels can occur only in the lateral direction. By doing so, the overlapping overhead due to the convolution with 2D kernels can be much smaller than the overlapping overhead due to the convolution with 3D kernels. Figure 4c shows that no overlapping overhead can be found for the sub-image convolution with 1D kernels. This pointwise convolution with 1D kernels does not suffer any overlapping overhead, because the kernel’s lateral dimension is as small as only one pixel. By doing so, the sub-image convolution with the pointwise 1D kernels does not make any overlapping between two neighboring sub-images.

**Figure 5:**
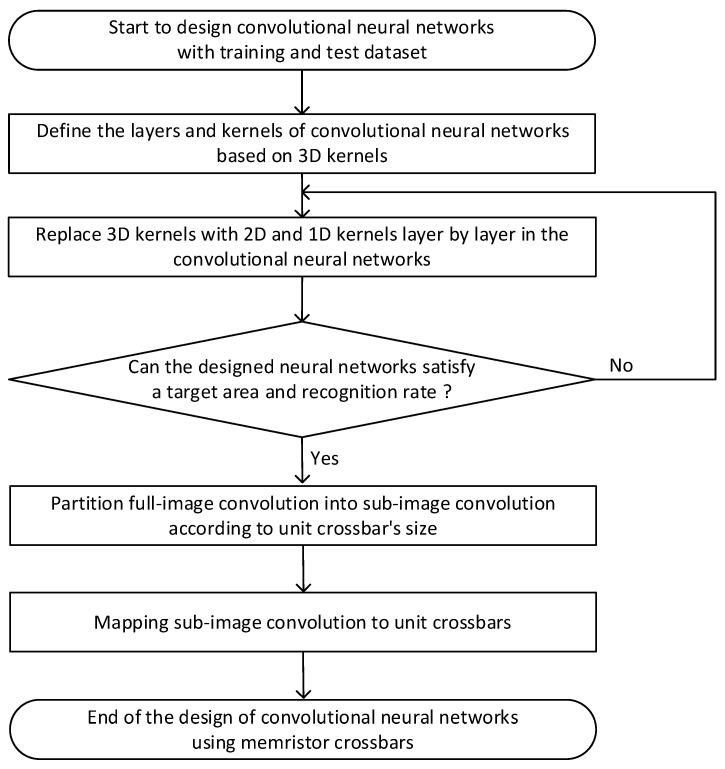
The area-efficient mapping method of convolutional neural networks to memristor crossbars using sub-image partitioning.

The comparison of 3D, 2D, and 1D kernels in Figure 4a–c indicates that the sub-image convolution with 3D kernels is worse than the 2D and 1D ones in terms of the overlapping overhead. Thus, to mitigate the overlapping overhead, it is better to use the 2D and 1D kernels more than the 3D ones in mapping the sub-image convolution to the memristor crossbars. Based on the analysis of sub-image partitioning and convolution schemes explained earlier, we propose an area-efficient mapping method of sub-image convolution to unit crossbars in this paper, as indicated in Figure 5. 

In the mapping method in Figure 5, we start the design of convolutional neural networks from the target dataset of training and testing. First, the layers and kernels used in the convolutional neural networks should be defined. Here, the 3D kernels are assumed to be used in the networks. After defining the neural network’s architecture, the convolution layers with 3D kernels can be replaced with 2D and 1D kernels layer by layer, in order to reduce the overlapping overhead caused from the sub-image convolution. As the 3D layers are replaced with the 2D and 1D ones layer by layer, the area of crossbars can be reduced, but the neural network’s performance is degraded. Based on the trade-off relationship between the neural network’s performance and crossbar’s area, the iteration goes on until the satisfaction of the target specification. In the following step, the full-image convolution is partitioned into the sub-image convolution according to the unit crossbar’s size. Then, the sub-image convolution can be finally mapped to the unit crossbars for performing the convolution physically.

## 3. Results

Table 1a,b show the convolutional neural networks using 3D kernels and 2D + 1D ones, respectively. The convolutional neural networks relying on 2D and 1D kernels rather than 3D kernels have been known as Depthwise Separable Neural Networks [25]. The neural networks in Table 1a,b are composed of 16 layers [26]. The fully connected layer is used at the final stage, where 1024 hidden neurons are connected to 10 output ones. In Table 1a, ‘CONV’ means the convolution layer by 3D kernels. ‘S1′ and ‘S2′ mean the stride numbers are 1 and 2, respectively. In Table 1b, ‘DW CONV’ and ‘PW CONV’ mean the convolution layers by 2D and 1D kernels, respectively.

**Figure 6:**
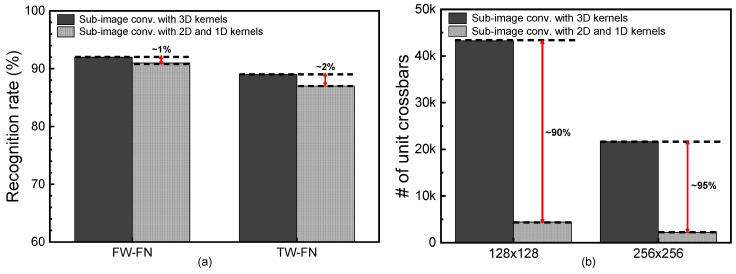
(**a**) The comparison of the recognition rate between the 3D and 2D+1D kernels. Here, the FW-FN means the floating-point weights and floating-point neurons used in the simulation. The TW-FN means the ternary weights and floating-point neurons. (**b**) The comparison of the number of unit crossbars used in the sub-image convolution between the 3D and 2D+1D kernels. Here, the unit crossbar’s size is assumed to be 128 × 128 and 256 × 256.

Figure 6a compares the neural network’s performance of the sub-image convolution between the 3D and 2D+1D kernels. The sub-image convolution with 3D kernels is used in the neural network’s architecture in Table 1a. The 2D and 1D kernels are used in the neural network’s architecture in Table 1b. In the neural network’s simulation, the CIFAR-10 dataset was used [22]. In the CIFAR-10 dataset, the number of training images is 50,000 and the number of test images is 10,000. The number of image categories is 10. Here, the simulation was performed by MATLAB and pytorch. In Figure 6a, the FW-FN means that both the synaptic weight and the neuron’s output are calculated with floating numbers. The TW-FN means the synaptic weight is represented with ternary values and the neuron’s output is calculated with floating numbers. For the FW-FN, the sub-image convolution with 3D kernels shows the recognition rate as high as 92% for the CIFAR-10 dataset. The convolutional network with 2D and 1D kernels shows the rate of 91%. The gap between the 3D and 2D+1D kernels is as small as 1%. For the TW-FN, the synaptic weight can be either -1, 0, or 1. In this case, the sub-image convolution with 3D kernels shows the rate of 89%. Similarly, the 2D and 1D kernels indicate the rate as high as 87%. The gap between the 3D and 2D+1D kernels is still as small as 2%.

Figure 6b compares the number of unit crossbars used in the 3D and 2D+1D kernels. Here, the unit crossbar’s size is assumed to be 128 × 128 and 256 × 256. When the sub-image convolution with 3D kernels is mapped to 128 × 128 unit crossbars, the number of unit crossbars becomes as large as 43,264. This large number of unit crossbars is due to the overlapping overhead of 3D kernels. As indicated in Figure 4a, the overlapping overhead of 3D kernels can be found both in the vertical and lateral directions. As the 3D kernel’s depth becomes deeper, the overlapping overhead in the vertical direction is increased more. Compared to the 3D kernels, the 2D kernels produce the overlapping overhead only in the lateral direction. The 1D kernels do not make the overlapping overhead. By doing so, the sub-image convolution with 2D and 1D kernels in Table 1b needs a much smaller number of unit crossbars than that with 3D kernels in Table 1a. Specifically, when the unit crossbar’s size is assumed to be 128 × 128, the sub-image convolution with 2D + 1D kernels can save the number of unit crossbars used in the neural networks by 90% compared to the sub-image convolution with 3D kernels. When the unit crossbar’s size becomes as large as 256 × 256, the percentage gap between the 2D + 1D and 3D kernels becomes larger, as shown in Figure 6b. For the 256 × 256 unit crossbar, the number of unit crossbars used in the sub-image convolution with 2D and 1D kernels is smaller by 95% than that of the sub-image convolution with 3D kernels.

Before ending the discussion of Figure 6a,b, it should be noted that the full-image convolution is not considered in the simulation of Figure 6a. This is because the full-image convolution without sub-image partitioning needs very large size crossbars, as explained in the previous section. In this case, the line resistance should become very large, too. The large line resistance can degrade the MAC calculation accuracy severely, as explained in Figure 2d. Thus, the recognition rate of the full-image convolution is much worse than that of the sub-image convolution, when the convolution operations are performed by memristor crossbars. This is the reason why the recognition rate of the full-image convolution is not considered as a baseline reference in Figure 6a.

**Figure 7:**
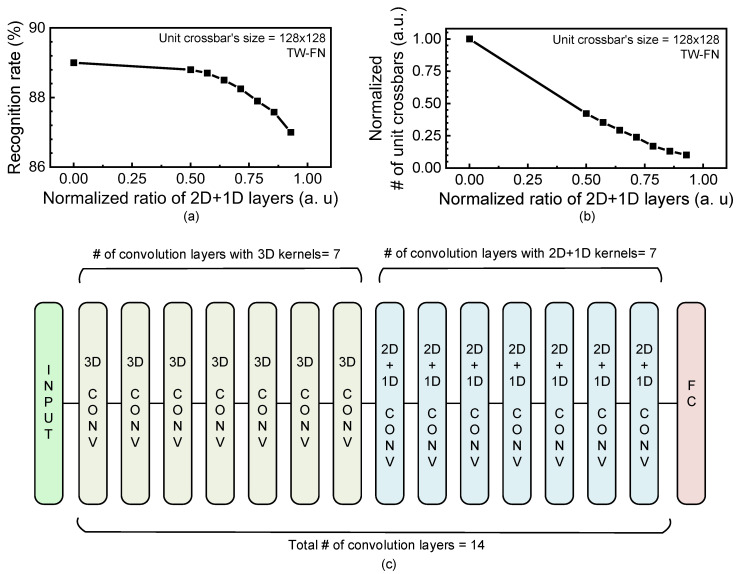
(**a**) The recognition rate with varying the ratio of 2D + 1D convolution layers from 0 to 1. The ratio of convolution layers with 2D + 1D kernels is calculated with the number of 2D + 1D layers divided by the total number of convolution layers. Here, it is assumed that the unit crossbar’s size is 128 × 128 and floating-point neurons and ternary synaptic weights are used in the neural network’s simulation. (**b**) The normalized number of unit crossbars used in the neural networks with varying the ratio of 2D + 1D convolution layers. (**c**) One example of the convolutional neural network’s architecture, when the numbers of 2D + 1D and 3D convolution layers are 7 and 7, respectively, among the total 14 convolution layers. The normalized ratio of 2D + 1D layers in this architecture is 0.5.

As explained in Figure 6a,b, the sub-image convolution with 3D kernels shows better recognition rate than the 2D + 1D kernels. However, the number of unit crossbars can be saved very much when the 2D + 1D kernels are used instead of the 3D kernels. To improve the neural network’s performance better, the convolution layers with 2D + 1D kernels can be combined with the layers with 3D kernels in one neural network. In Figure 7a,b, the recognition rate and the normalized number of unit crossbars are obtained with varying the ratio of convolution layers with 2D + 1D kernels among all the neural network’s layers. Here, the normalized ratio shown in the *x*-axis of Figure 7a and b is calculated with the number of 2D + 1D layers divided by the total number of convolution layers. As shown in Figure 7a, the recognition rate seems little changed until the normalized ratio of 2D + 1D layers becomes as small as around 0.5. In Figure 7b, when the normalized ratio of 2D + 1D layers is around 0.5, the number of unit crossbars can be almost half the normalized ratio = 0. Here, the normalized ratio = 0 means that the 2D + 1D convolution layer is not used in the networks. Figure 7c shows one example of the convolutional neural network’s architecture, when the numbers of 2D + 1D and 3D convolution layers are 7 and 7, respectively, among the total 14 convolution layers. In Figure 7c, the normalized ratio of 2D + 1D layers can be calculated with 0.5. One thing to note here is that the 2D + 1D convolution layers should be used in latter stages in the neural network’s architecture, as shown in Figure 7c, to achieve a better recognition rate.

Here, it should be noted that the MATLAB and pytorch simulation results were verified by the circuit simulation results of CADENCE SPECTRE. Because the circuit simulation is much slower than the MATLAB and pytorch, in this paper, only a part of the hybrid circuit composed of memristors and CMOS devices is simulated for MNIST testing vectors. From the simulation, it was observed that the MAC result calculated from the circuit simulation is the same with the MATLAB and pytorch simulation. Here, the circuit simulation was performed by CADENCE SPECTRE software [27]. In the simulation of the hybrid circuit of memristors and CMOS devices, SAMSUNG 65nm SPICE parameters are used. For simulating memristors, the Verilog-A model presented in the previous reference is used [28]. 

**Figure 8:**
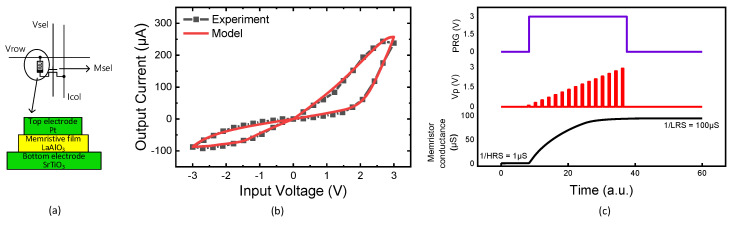
(**a**) The memristor circuit with a 1T(transistor)-1R(memristor) cell. The memristor is composed of a top electrode, memristive film, and bottom electrode. (**b**) The memristor’s butterfly curves from the experimental data (black box) and Verilog-A model (red line). (**c**) The simulated waveforms of memristor circuit [28].

Figure 8a shows a memristor circuit composed of 1T(transistor)-1R(memristor). Here, ‘Vrow’ means a row voltage applied to the crossbar. ‘Icol’ is a column current that calculates the MAC result. ‘Msel’ is a selector made of the CMOS transistor. As mentioned earlier, we used SAMSUNG 65nm CMOS process parameters in the circuit simulation. In Figure 8a, the memristor used in the circuit simulation was modeled using Verilog-A [7]. In the bottom of Figure 8a, the memristor’s top electrode is made of platinum. The memristive film is LaAlO_3_. The bottom electrode is SrTiO3. A butterfly curve from the device in Figure 8a is shown in Figure 8b [7,29]. The block box and red line in Figure 8b indicate the experimental measured data and the Verilog-A model, respectively, in Figure 8b. The High-Resistance State (HRS) and Low-Resistance State (LRS) measured in Figure 8b are around 1MΩ and 10KΩ, respectively [28], when the read voltage is as large as 1V. Considering a transistor as the selector, when the transistor is on, the effective resistance considering both LRS and the transistor’s ON resistance can be as small as 26.3 KΩ. The effective resistance due to HRS and the transistor’s ON resistance is very similar with HRS, because the ON resistance is much smaller than HRS. Thus, if the transistor’s ON resistance is comparable to LRS but much smaller than HRS, the MAC calculation accuracy of 1T-1R crossbars cannot be degraded. When the transistor is turned off, its OFF resistance is much larger than HRS. By doing so, the sneak leakage for unselected cells can be negligibly small in memristor crossbars.

Figure 8c indicates that the memristor modeled by the Verilog-A model in Figure 8b can be programmed by applying voltage pulses [7,30]. Here, the upper row in Figure 8c shows an enable signal of the memristor’s programming. If ‘PRG’ is high, programming pulses generated from the pulse generator circuit are delivered to the memristor. If ‘PRG’ is low, the programming pulses are blocked from being delivered to the device. In the middle row in Figure 8c, ‘VP’ represents programming pulses with their amplitudes increased gradually. The pulse amplitude modulation was used in the circuit simulation in order to accelerate the programming speed. The lower row in Figure 8c shows that the memristor’s conductance changed according to the programming pulses applied to the device. As the programming pulses are delivered to the device, the memristor’s conductance can be changed from 1/HRS to 1/LRS, as shown in Figure 8c.

From the circuit simulation using CADENCE SPECTRE, the LRS read current is estimated around 38 μA per cell, when the read voltage is 1V and a 1T-1R crossbar is used. If the memristor has HRS, the read current can be as small as 1 μA per cell. For the memristors not selected, the memristor’s current can be negligibly small, because the selector’s OFF resistance is larger than HRS by three orders of magnitude. For the transient characteristic of memristors, the programing and read pulse widths were measured around ~100ns [30]. The power consumption of neural networks is estimated using the hybrid circuit of memristors and CMOS devices for 10,000 MNIST test vectors. Here, the input, hidden, and output neurons are 784, 250, and 10, respectively, in the neural networks. The neural networks are implemented with memristor crossbars of 1T-1R, as shown in Figure 8a. The simulation indicates that the crossbar’s current consumption is as large as 11.9 mA on average, when the read pulse width is 100 ns and the operation frequency is 1 MHz. Here, LRS and HRS are assumed 10 KΩ and 1 MΩ, respectively, as indicated in Figure 8a.

## 4. Conclusions

Memristor crossbars can be very useful for realizing edge-intelligence hardware, because the neural networks implemented by memristor crossbars can save significantly more computing energy and layout area than the conventional CMOS digital circuits. One of the important operations used in neural networks is convolution. For performing the convolution by memristor crossbars, the full image should be partitioned into several sub-images. By doing so, each sub-image convolution can be mapped to small-size unit crossbars, where the crossbar’s size should be defined as a fixed size such as 128 × 128 or 256 × 256 to avoid the line resistance problem of large-size crossbars. 

To propose the area-efficient mapping method of sub-image convolution to unit crossbars, the various convolution schemes with 3D, 2D, and 1D kernels were investigated and compared in terms of the neural network’s performance and overlapping overhead in this paper. Based on the investigation and comparison, the 2D+1D kernels indicated that they could perform the convolution using a much smaller number of unit crossbars with less rate loss than the 3D kernels. When training and testing the CIFAR-10 dataset, the mapping of sub-image convolution of 2D+1D kernels to unit crossbars could save the number of unit crossbars almost by 90% and 95%, for the unit crossbar’s size of 128 × 128 and 256 × 256, respectively, compared with the 3D kernels. On the contrary, the rate loss of 2D+1D kernels was less than 2%. To minimize the rate loss more, the 2D+1D kernels could be combined with 3D kernels in one neural network. When the normalized ratio of 2D+1D layers is around 0.5, the neural network’s performance indicated very little rate loss using only half of unit crossbars compared to the normalized ratio of 2D+1D layers of zero.

## Figures and Tables

**Table 1:** (a) The neural network’s architecture of sub-image convolution using 3D kernels. (b) The neural network’s architecture of sub-image convolution using 2D and 1D kernels.

Layer #	Type/Stride	Kernel Shape	Input Size
**(a)**			
1	CONV/S1	(3 × 3 × 3) × 32	32 × 32 × 3
2	CONV/S1	(3 × 3 × 32) × 64	32 × 32 × 32
3	CONV/S2	(3 × 3 × 64) × 128	32 × 32 × 64
4	CONV/S1	(3 × 3 × 128) × 128	16 × 16 × 128
5	CONV/S2	(3 × 3 × 128) × 256	16 × 16 × 128
6	CONV/S1	(3 × 3 × 256) × 256	8 × 8 × 256
7	CONV/S2	(3 × 3 × 256) × 512	8 × 8 × 256
8 ~ 12	CONV/S1	(3 × 3 × 256) × 512	4 ×4 × 512
13	CONV/S2	(3 × 3 × 512) × 1024	4 ×4 × 512
14	CONV/S1	(3 × 3 × 1024) × 1024	2 × 2 × 1024
15	AVG POOL/S2	(2 × 2)	2 × 2 × 1024
16	FC	(1024 × 10)	1024
**(b)**			
1	CONV/S1	(3 × 3 × 3) × 32	32 × 32 × 3
2	DW CONV/S1	(3 × 3 × 1) × 32	(32 × 32 × 1) × 32
	PW CONV/S1	(1 × 1 × 32) × 64	32 × 32 × 32
3	DW CONV/S2	(3 × 3 × 1) × 64	(32 × 32 × 1) × 64
	PW CONV/S1	(1 × 1 × 64) × 128	16 × 16 × 64
4	DW CONV/S1	(3 × 3 × 1) × 128	(16 × 16 × 1) × 128
	PW CONV/S1	(1 × 1 × 128) × 128	16 × 16 × 128
5	DW CONV/S2	(3 × 3 × 1) × 128	(16 × 16 × 1) × 128
	PW CONV/S1	(1 × 1 × 128) × 256	8 × 8 × 128
6	DW CONV/S1	(3 × 3 × 1) × 256	(8 × 8 × 1) × 256
	PW CONV/S1	(1 × 1 × 256) × 256	8 × 8 × 256
7	DW CONV/S2	(3 × 3 × 1) × 256	(8 × 8 × 1) × 256
	PW CONV/S1	(1 × 1 × 256) × 512	4 × 4 × 256
8 ~ 12	DW CONV/S1	(3 × 3 × 1) × 512	(4 × 4 × 1) × 512
	PW CONV/S1	(1 × 1 × 512) × 512	4 × 4 × 512
13	DW CONV/S2	(3 × 3 × 1) × 512	(4 × 4 × 1) × 512
	PW CONV/S1	(1 × 1 × 512) × 1024	2 × 2 × 512
14	DW CONV/S1	(3 × 3 × 1) × 1024	(2 × 2 × 1) × 1024
	PW CONV/S1	(1 × 1 × 1024) × 1024	2 × 2 × 1024
15	AVG POOL/S2	(2 × 2)	2 × 2 × 1024
16	FC	(1024 × 10)	1024
